# A Handbook of Hospital Practice, or an Introduction to the Practical Study of Medicine at the Bedside

**Published:** 1861-07

**Authors:** 


ART. VIII.—*A Handbook of Hospital Practice, or an Introduction to the Practical Study of Medicine at the Bedside.* By ROBERT D. LYONS, M.D., Physician to Jervis Street Hospital, Dublin. 12mo., pp. 186. New York: Samuel S. & William Wood, 1861.

THERE are more medical men, we fear, who know how to make observations at the bedside, than comprehend the uses to which their investigations may be applied. The tendency is to collect symptoms and histories of cases without, in many instances, rightly understanding the connection between them, or perhaps without possessing the preliminary knowledge essential to the correct interpretation of the facts collected. Many a discovery, interesting to the profession, might long since have been made, had intelligent observers recorded their investigations, and followed them up from the facts deemed worth retaining at the time of their occurrence.

The work before us is intended to guide the student to the best modes of observing phenomena in the clinical examination of patients, and of recording them for future reference. The author at the outset furnishes the reader with "a skeleton map of the countries he is about to explore," a tabular view of the diseases to which the human frame is liable. The nosology proposed by Dr. Farre, and employed in the registration of deaths in England, is that adopted by the author, and is considered perhaps more simple, comprehensive, and satisfactory than any other. Without entering into the minutiæ of classes and orders, and their subdivisions, we may state that all diseases are divided by him into four classes:—1. Zymotic diseases. 2. Constitutional diseases. 3. Local diseases. 4. Developmental diseases. And what a catalogue a list of this kind embraces—every disease which gives us pain, and anxiety, and dread summed up in one long table of eight columns, with sometimes almost unpronounceable Latin names added to them!

The subject of Preliminary Examination of a Patient embraces a careful summary and detailed description of bedside practice in the apparently minor but eminently important facts which may be learned by the exercise of the senses of sight, hearing, smell, and touch, before a single interrogatory is addressed to the patient. The author dwells upon the essential points of a correct diagnosis gleaned in this way, but the practitioner ought at once to learn to study such details himself without appealing to books for assistance. At the outset, however, a guide to medical diagnosis may be a valuable companion to him, and as such, the instruction obtained from a work like that of Dr. Lyons might be replete with useful hints.

The order proposed by the author for the examination and recording of the history of a case, and for the exploration of the condition of the several functions and organs, is the following: I. The antecedent health and diseases, precise date, first symptoms and progress of present disease till first examination by the reporter of the case. II. The symptoms and signs presented under the following heads: General moral and physical condition; state of cerebral functions and organs; state of the pulse, and other general phenomena of the circulation; state of respiration; of the general cutaneous system, etc. III. Therapeutic applications. IV.

Daily progress of the case. V. Termination of the case. The arrangement is certainly simple enough, and no confusion could possibly occur in such a record.

The remaining special divisions of the Clinical Examination of Patients refer to the true modes and uses of practicing auscultation and percussion, the importance, as diagnostic symptoms, of expectoration, hemorrhages, the examination of the urine, etc. One of the most valuable chapters in the work is that devoted to post-mortem examinations, each portion of the body being taken up for consideration, and much practical information being afforded in regard to dextrous manipulations and points of delicate management which so often tell to the credit or the discredit of the physician. The writing of prescriptions, a glossary, and forms for recording cases occupy the remainder of the work.

